# Implementation Strategies Applied in Communities Matching Process (ISAC Match): Expanded Guidance and Case Study

**DOI:** 10.1186/s13012-025-01456-1

**Published:** 2025-10-17

**Authors:** Laura E. Balis, Taren Massey-Swindle, Shelly Palmer, Emily Shaw, Shelby Jones-Dozier, Michelle Grocke-Dewey

**Affiliations:** 1 Center for Nutrition & Health Impact, Omaha, NE USA; 2 Department of Pediatrics, University of Arkansas Medical Sciences, Little Rock, AR USA; 3 Arkansas Children’s Nutrition Center, Little Rock, AR USA; 4 Arkansas Children’s Research Institute, Little Rock, AR USA; 5 Montana State University Extension, Bozeman, MT USA; 6 Department of Human Development and Community Health, Montana State University, Bozeman, MT USA

**Keywords:** Implementation strategies, Implementation outcomes, Tailoring, Contextual inquiry, Health equity, Integrated research-practice partnerships, Pragmaticism, Community settings, Cooperative Extension

## Abstract

**Background:**

Implementation strategies are methods or techniques to improve the adoption, implementation, sustainment, and scale-up of evidence-based interventions. Limited guidance exists on feasible processes for selecting and tailoring implementation strategies in community (non-clinical) settings. The Implementation Strategies Applied in Communities (ISAC) compilation includes a pragmatic matching process to accompany the compilation (ISAC Match). This study expands on ISAC Match by providing additional detail and potential approaches to complete the four-step matching process, including a case study from work in a state Cooperative Extension System.

**Implementation Strategies Applied in Communities Matching Process (ISAC Match):**

ISAC Match is intended to be applied within integrated research-practice partnerships or similar models. Before beginning the ISAC Match process, participants should have identified a new or existing evidence-based intervention they are interested in integrating (or improving the integration of) and have the power and scope to influence implementation. ISAC Match includes four steps: 1) reviewing available information on evidence-based intervention integration and conducting contextual inquiry, if needed, to understand barriers and facilitators; 2) identifying existing implementation strategies used in the implementing organization, 3) using recommended guidance tools to select relevant implementation strategies to overcome barriers and capitalize on facilitators; and 4) tailoring strategies to fit within the setting they will be used in. These steps are completed with health equity considerations in mind to ensure that implementation strategies are designed to improve adoption, implementation, and maintenance in ways that seek to narrow existing health disparities. To illustrate the use of ISAC Match, this study applied the four-step ISAC Match process to select and tailor implementation strategies to increase Montana State University Extension Agents’ adoption of built environment approaches that facilitate physical activity.

**Conclusions:**

The ISAC match process was developed to apply to community settings because of a lack of guidance on rapid, relevant methods for selecting and tailoring implementation strategies to overcome barriers and capitalize on facilitators. Future work is needed to determine whether the ISAC match process is more efficient and whether results are more impactful than other matching processes that are less specific to community settings.

**Supplementary Information:**

The online version contains supplementary material available at 10.1186/s13012-025-01456-1.

Contributions to the literature

- Guidance is needed for a practical, rapid process for selecting and tailoring implementation strategies in community settings.
- We present ISAC Match, a four-step process designed for integrated research-practice partnerships to collaboratively conduct contextual inquiry, if needed, identify existing implementation strategies, and select and tailor relevant implementation strategies.
- We provide a case study using ISAC Match to identify implementation strategies to support the adoption of built environment approaches to increase physical activity levels.

## Background

Implementation strategies are methods or techniques to improve the adoption, implementation, sustainment, and scale-up of evidence-based interventions (EBIs) [1–4]. The process of selecting and tailoring implementation strategies has previously been described as a “black box” with limited details [5]. Multiple methods have been suggested to make the selection and tailoring process more systematic, yet existing methods typically require expert consultation and specific training [5] or are not specific to community settings [6]. For example, the Consolidated Framework for Implementation Research – Expert Recommendations for Implementing Change (ERIC) match tool is a promising approach for generating a list of potential strategies matched to selected barriers [6]. However, in other studies it has been found difficult to use in community settings due to the clinical setting language in the ERIC compilation and the high number of strategies generated, which can be difficult and timely to prioritize [7]. With this lack of pragmatic and community-friendly processes, the status quo in community settings is selecting implementation strategies without understanding contextual factors [8–10]. This can lead to strategies such as “training and hoping” [11, 12] that are not adequate to overcome organizational or external (e.g., community) barriers.

Community-engaged approaches to selecting implementation strategies are essential for understanding local context, selecting relevant strategies, and coproducing implementation strategies that are shaped by delivery system values, resources, and infrastructure [13]. Recently, the Implementation Strategies Applied in Communities (ISAC) compilation was developed to provide a list of implementation strategies used in community settings (e.g., social services, faith-based, education, and non-clinical public health organizations) [14]. ISAC was developed to address the need for implementation strategies appropriate for community settings, as existing compilations, such as the Expert Recommendation for Implementing Change (ERIC) compilation, were developed in clinical settings and can be difficult to apply in community settings [15]. The ISAC study identified 40 implementation strategies used in community settings to integrate primary prevention EBIs; 24 (60%) had similar content to ERIC strategies with community setting-specific language while 16 strategies (40%) were unique. Researchers may find benefit in using ISAC and/or ERIC depending on their intervention setting. For example, researchers working on community-clinical linkage interventions (e.g., Food is Medicine) may require strategies from each compilation [16].

In addition, because of the challenges associated with using implementation strategies in community settings (e.g., lower resources, lack of implementation-specific staff), a need for guidance on selecting strategies was identified. As part of the ISAC study, researchers developed a pragmatic matching process to accompany the compilation: the Implementation Strategies Applied in Communities Matching Process (ISAC Match). ISAC Match makes the selection process more practical and rapid. Moreover, it uses a strength-based approach (i.e., considering both barriers and facilitators) in the decision-making process, which is recommended in community public health settings.

In this debate, we expand on ISAC Match by providing additional detail and potential approaches to complete each of its four steps. We also include a case study from our work identifying implementation strategies to increase the adoption of built environment approaches in a state Cooperative Extension System. The overall goal of the debate is to provide an actionable tool for both researchers and practitioners to understand local context and select and tailor relevant implementation strategies.

## Implementation Strategies Applied in Communities Matching Process (ISAC Match)

ISAC Match is intended to be applied within partnerships between researchers and practitioners who have the power and scope to influence implementation. Ideally, this is accomplished through an integrated research-practice partnership (IRPP) or similar model that equally values the contributions of both researchers and practitioners (i.e., those who are charged with implementing EBIs) [13]. Cohesion is foundational to IRPPs; they succeed when both practice needs (e.g., integrating a new EBI) and research needs (e.g., studying how to best integrate the EBI) are met [17]. IRPPs have been used to collaboratively select, adapt, implement, and evaluate both evidence-based interventions and implementation strategies [13, 18].

Briefly, to generate translational solutions, the IRPP members work through a collaborative decision-making process that includes problem prioritization, strategy selection, strategy adaptation, integration trials, evaluation, and decision-making [13]. Each step of the process includes all IRPP members (both researchers and practitioners). While IRPP composition may vary depending on the setting and key individuals involved (e.g., both decision-makers and implementers in a practice setting) [13], smaller groups (e.g., less than 10 members) are likely to be more effective [19, 20]. First, prior to beginning ISAC Match, IRPP members identify a new or existing EBI to be integrated (or better integrated, e.g. by addressing challenges in fidelity). The ISAC Match process is conducted during the problem prioritization, strategy selection, and strategy adaptation phases. The pre-integration trial, evaluation, and decision-making phases are completed following the ISAC Match process. We adapted Fig. 1 from Estabrooks et al. [13] to detail how and when ISAC Match steps are completed within the IRPP process and highlight potential steps to be completed following ISAC Match.

**Fig. 1:**
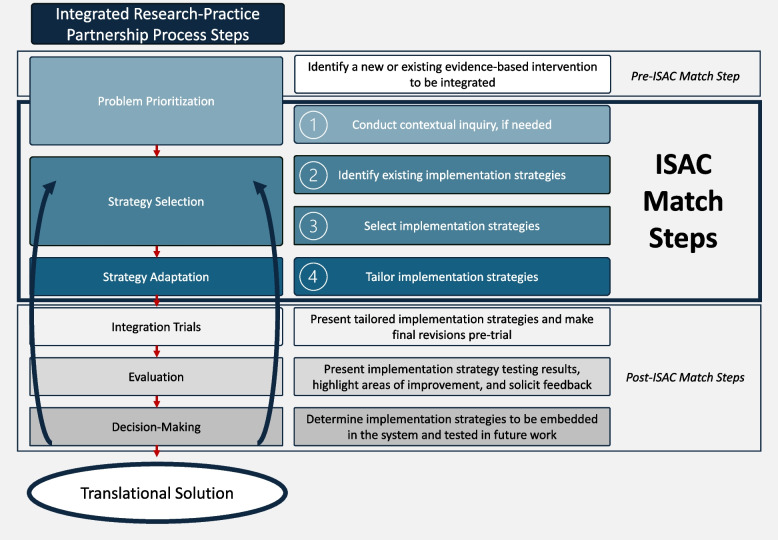
ISAC Match Steps completed as part of the Integrated Research-Practice Partnership process [13]

ISAC Match includes four steps: 1) reviewing available information on EBI integration and conducting contextual inquiry, if needed, to understand barriers and facilitators; 2) identifying existing implementation strategies used in the practice setting, 3) using recommended guidance tools to select relevant implementation strategies to overcome barriers and capitalize on facilitators; and 4) tailoring strategies to fit within the setting [14]. These steps are completed with health equity considerations in mind to ensure that implementation strategies are designed to improve adoption, implementation, and maintenance in ways that do not contribute to and seek to narrow existing health disparities. General guidance is given in the following section, with specific data collection approaches detailed in Table 1.

**Table 1:** Steps and Potential Approaches for the Implementation Strategies Applied in Communities Matching Process (ISAC Match)

Step	Potential approaches
1. Conduct contextual inquiry, if needed	*Determine if contextual inquiry is needed:* • Review available information on EBI integration • Include both peer-reviewed and grey literature (e.g., practice reports) to identify practice-base evidence [21] *If additional contextual inquiry is needed, use rapid methods:* • Rapid deductive qualitative approaches [22–24] • Rapid ethnography [25] • Brainwriting premortem approaches [26, 27] *If there is a need to prioritize barriers to address:* • Card sorting activity [28] • Rate barriers by changeability and importance (survey or 2 × 2 grid) [29–31]
2. Identify existing implementation strategies	• Engage with practitioners and review practice materials • Facilitate a discussion to identify existing strategies
3. Select implementation strategies	• Use ISAC guidance tool to select implementation strategies by determinant framework level [14] • Use ISAC guidance tool to select implementation strategies by implementation outcomes (RE-AIM dimension) [14] *If there is a need to prioritize implementation strategies to test:* • Rate strategies by feasibility and importance (survey or 2 × 2 grid) [29–31] • Assign 100 priority points to potential strategies [29] • Nominal group techniques [32]
4. Tailor implementation strategies	• Use a brainwriting premortem process to identify potential reasons strategies would fail [26] • Nominal group techniques [32] • Liberating structures [33, 34]

### Step 1. Conduct contextual inquiry, if needed

Contextual inquiry (also called needs assessments or formative evaluation) to understand local implementation determinants is key to selecting implementation strategies [1, 5, 35–37]. However, there have been calls in implementation science for this process to be conducted more quickly and not contribute to translational lag time, as it often takes one to two years per study [38, 39]. For example, when barriers and facilitators to implementing certain interventions have already been investigated, researchers and practitioners could review this information and decide if additional contextual inquiry is needed (e.g., if the setting or priority population is novel) [38, 39]. In cases when additional contextual inquiry is needed, it could be done rapidly through confirming whether barriers and facilitators identified in the literature are applicable to invested parties (e.g., implementers, administrators) in new settings [38, 39]. If additional contextual inquiry is not needed, the IRPP members can move on to step 2.

If little evidence exists on contextual factors relevant to the EBI, contextual inquiry may need to be completed. This can be done through research designed to understand implementation context, for example, using the Consolidated Framework for Implementation Research (CFIR) or other determinant frameworks [40–44]. Ideally, to continue to speed the translation of research to practice, this is done through rapid approaches such as rapid deductive qualitative methods or rapid ethnography [22, 38]. Alternatively, teams could focus the research question on which implementation outcomes will be challenging to achieve and could use a brainwriting premortem approach (i.e., discussing why implementation efforts might fail) based on the RE-AIM (reach, effectiveness, adoption, implementation, maintenance) framework [26, 27].

Contextual inquiry methods may result in the discovery of multiple barriers, in which case teams may need to make decisions on which to address. This can be done through ranking and prioritizing methods such as card sort activities [28, 45] (e.g., into low, medium, and high priority groupings) or modified conjoint analysis (rating strategies by changeability and importance through a survey or by placing sticky notes with each barrier on a 2 × 2 grid poster board) [29–31].

### Step 2. Identify existing implementation strategies

Practitioners in community settings are often using implementation strategies, although they may not be referred to as such. For example, in the National Cooperative Extension System, which serves every U.S. state and territory by bringing land-grant university research to community members, state-level specialists (university faculty) typically support county-based personnel who deliver EBI to community members [46, 47]. This support may be similar to facilitation or technical assistance but may not have been defined or operationalized this way. Additionally, practitioners may have created implementor resources such as program guides or implementation blueprints that serve as implementation strategies [7]. Understanding which strategies are already in place can lead to refining or complementing versus developing redundant or competing strategies [48]. For example, existing program guides could be tailored to include specific guidance to overcome barriers, or could be used to supplement technical assistance. To identify existing supports, we recommend reviewing practice materials or facilitating a discussion to identify existing implementation strategies. The discussion could include thinking through past barriers to adopting, implementing, and maintaining EBI and what organizational supports or resources were helpful in overcoming challenges.

### Step 3. Select implementation strategies

Next, decisions need to be made on which of all available implementation strategies may help practitioners overcome barriers and capitalize on facilitators. In some studies, this is done by reviewing all strategies in a compilation – for example, the seminal ERIC implementation strategies – and discussing which may be applicable. To make this process more rapid and focus on relevant strategies, we suggest researchers narrow the options for practitioners to discuss (e.g., versus discussing all 73 ERIC strategies or all 40 ISAC strategies). Depending on the methods used in Step 1, this can be done through selecting potential strategies by the barriers they are likely to overcome (by determinant framework level) or the implementation outcomes they are likely to improve (reach, adoption, implementation, or maintenance) [14]. Guidance tools for both options are included in the ISAC compilation: one includes potential strategies indicated by determinant framework level (e.g., there are 11 ISAC strategies identified to overcome individual-level barriers); the other includes potential strategies by RE-AIM framework dimension (e.g., 13 strategies identified to improve EBI maintenance) [14].

In addition, strategies that have already been matched to barriers and tested in similar settings may be present. If so, as teams confirm whether the barriers and facilitators found in the literature are relevant and identify any new barriers and facilitators (in Step 1), the previously tested implementation strategies could be selected for consideration and integrated with implementation strategies selected through the ISAC guidance tools. To consider health equity and differences in resources between settings, strategies should ideally be selected at multiple levels (e.g., the inner setting and outer settings of the CFIR) to avoid focusing only on improving individual skills or knowledge without considering existing systems and structures [12]. Additionally, implementation facilitators should be considered as implementation strategies are being selected. For example, if selecting a strategy to overcome an already identified barrier of funding, teams could consider an identified facilitator of existing connections and partnerships, and then select a strategy to leverage shared resources in community coalitions.

Finally, implementation strategies will likely need to be prioritized to select a feasible number for testing within the constraints of a project or grant. Similar methods as those used to prioritize barriers in Step 1 can be used [45], including ranking strategies by feasibility and importance [29–31], assigning 100 priority points to potential strategies [29], or nominal group techniques that consider all participants’ views equally to generate priorities and reach consensus [32]. Through the selected decision-making strategies, we recommend discussing results to ask partnership members to articulate why they believe specific strategies will work. This can help the team ensure there is mutual understanding of what a strategy entails and what outcomes it would lead to. Practitioners’ explanations could also help identify the selected implementation strategies’ mechanisms of action to ensure they are grounded in theory and can be tested in implementation trials.

### Step 4. Tailor implementation strategies

Implementation strategies need to be tailored to ensure they align with the setting in which they are used. The process of tailoring considers the actors, targets, temporality, and dose of the strategy [49] that is needed to improve implementation outcomes in the specific setting for the selected intervention while considering assets, culture, available resources and other contextual factors important for health equity [44]. For example, tailoring could include changing how often an implementation strategy is delivered (dose) or who delivers it (actor) [49]. The process of tailoring implementation strategies, as with other steps in the ISAC process, is completed with practitioners. Multiple methods are available [45], including a brainwriting premortem process focused on reasons why selected strategies could fail [26], nominal group techniques [32], or liberating structures (practical methods to enhance group decision making) [33, 34]. Once implemented, those involved in the IRPP are encouraged to track implementation strategy modifications over time to aid understanding of what is adapted, why, and how, and ultimately increase understanding of successful implementation strategies. The Framework for Reporting Adaptations and Modifications to Evidence-based Implementation Strategies (FRAME-IS) [50], Longitudinal Implementation Strategy Tracking System, and other tools are available to support this process [51].

## Case Study

**Fig. 2:**
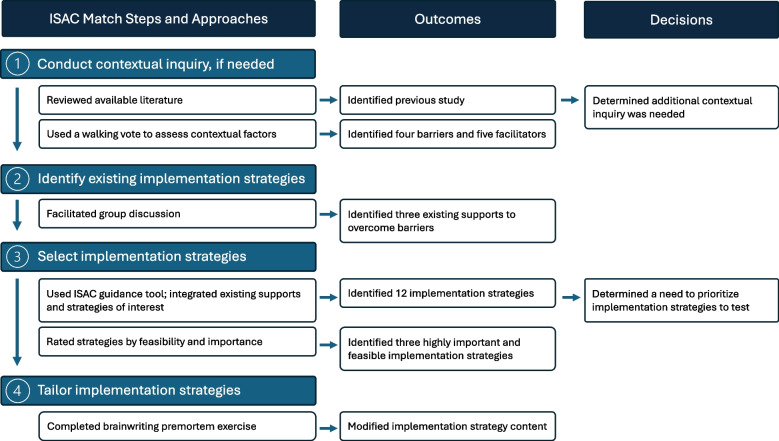
Approaches used for each ISAC Match step in the case study, with outcomes and decisions used to inform further data collection

To illustrate the use of ISAC Match, we describe application of the four-step process to select and tailor implementation strategies to increase the adoption of built environment approaches within the Montana State University Extension System (Fig. 2). Built environment approaches make it safer and easier for people across the life span to walk, bicycle, or wheelchair roll, and mitigate barriers to physical activity by making the healthy choice the default choice [52–54]. The National Cooperative Extension System has historically focused on individual-level educational programming; however, because of recent mandates and shifts in priorities, the system is now poised to implement built environment approaches [8, 55–57]. However, barriers exist [58–60], and relevant implementation strategies are needed to improve adoption rates.

As described below, strategies were selected and tailored through an IRPP approach. The IRPP included nine members: seven Extension Agents (county-based field faculty who select and implement interventions that meet identified county needs and align with their skills and interests [61–66]), including SJD, and two researchers (the principal investigator, LB, who had previous experience in the Extension system, and co-investigator, MGD, who serves as an Extension Specialist). The team was supported by a project manager (SP) and research associate (ES). The IRPP members engaged in two decision-making sessions to collaboratively select and tailor implementation strategies to address the identified barriers and capitalize on facilitators [13]. The first session, held one month after project initiation, was a six-hour in-person project kickoff meeting, which included team introductions, project overview and timeline, a decision-making warm-up activity, overviews of built environment approaches and implementation strategies, and Steps 1–3 of the ISAC Match process. The second session, conducted two months later, was a virtual two-hour meeting conducted to complete Step 4.

### Step 1. Conduct contextual inquiry, if needed

The researchers had previously completed a brief contextual inquiry study (< 10 min survey) to identify Montana Extension Agents’ barriers and facilitators to implementing built environment approaches [58]. However, as the study had been completed over two years before the project was initiated and staff turnover had occurred, the researchers determined that additional brief contextual inquiry was needed to confirm if the results of the previous study were still relevant and identify any new barriers and facilitators.

Thus, the researchers reviewed available literature on barriers and facilitators to built environment approaches in state Extension systems [59, 60] and developed a list, organized by CFIR level (outer, inner, individual/intervention), of barriers and facilitators that had been identified in state Extension systems and the previous study that included Montana [58]. During the first IRPP session, the group used a “walking vote” to review each potential barrier and facilitator: after each was read aloud, Agents were asked to walk to one side of the room or the middle to indicate whether each factor was a barrier, facilitator, or neither.

All seven Agents participated in the walking vote. At the *outer setting level*, community members’ attitudes (i.e., that physical activity may not be the norm) was a barrier. Community support, partnerships and connections, and the existing built environment were facilitators. At the *inner setting level***,** resources (i.e., funding), communication (with Extension colleagues in other initiative areas, such as Community Vitality), and access to knowledge and information were barriers; there were no facilitators. At the *individual and intervention levels*, there were no barriers; motivation and opportunity were facilitators as Agents were interested in built environment approaches and felt they were within their scope of work.

### Step 2. Identify existing implementation strategies

Next, to determine what implementation strategies were already in place, the researchers led a group discussion with the prompt “What implementation strategies, resources, or supports are already in place to overcome barriers?” All seven Agents participated in the discussion; they shared three existing supports relevant to overcoming the noted barriers: 1) *tailor recruitment strategies:* skills in marketing through the Extension Specialist and an administrative assistant*,* 2) *increase demand:* individual-level physical activity programming, such as Walk with Ease and arthritis programs that could be used to influence community members’ attitudes around physical activity and the built environment, and 3) *build partner relationships:* connections with the Montana Department of Public Health and Human Services’ prevention programs, which could be used to form strategic partnerships to both invest in and promote the chosen built environment approaches.

### Step 3. Select implementation strategies

The previous contextual inquiry study also included an item asking respondents to rank interest in potential implementation strategies. These strategies were reviewed through group discussion to determine whether they were still relevant to address barriers. The group selected one of the strategies, *leverage funding sources*, as relevant, and shared that they were interested in both obtaining mini-grants and receiving assistance to apply for larger grants.

Next, to overcome the barriers identified in Step 1, the group reviewed a list of potential implementation strategies organized by CFIR level based on the ISAC guidance tool. This included 13 implementation strategies to overcome outer setting barriers and 10 to overcome inner setting barriers. The group reviewed strategies at each level through group discussion. The researchers provided the names and definitions of potential strategies and asked practitioners whether they thought each would be useful to overcome the identified barriers. The researchers and practitioners recorded 10 strategies deemed potentially useful and consolidated with the three existing strategies identified in Step 2 (e.g., *build partner relationships*) and the strategy of interest from the previous study (*leverage funding sources*). This resulted in 12 strategies selected to assess for importance and feasibility.

Agents completed an online Qualtrics survey to rate each of the 12 potential implementation strategies (described in practitioner-friendly language) for importance and feasibility on 5-point Likert scales (1 = relatively unimportant/not at all feasible, 5 = extremely important/extremely feasible) [30, 31]. Data were analyzed and shared with the team during the IRPP session to determine highly important and feasible implementation strategies to prioritize for discussion. Mean importance and feasibility ratings were analyzed through Qualtrics and viewed through the Qualtrics data dashboard. The research team identified strategies with mean ratings of at least 4.0 on both scales (very important/very feasible).

Six Agents completed the importance and feasibility survey; one was unavailable during this portion of the session. Five strategies had mean ratings of 4.0 or higher for both feasibility and importance (Table 2). See Additional file 1 for raw data. The research team shared these highly rated strategies with the Agents and asked for additional input on how they envisioned each strategy being used. Based on Agents’ feedback, the IRPP members eliminated *use technology for evaluation* as an implementation strategy, as Agents clarified that they were most interested in assistance with compiling statewide results of built environment approaches to demonstrate impact. The research team determined that this was not an implementation strategy likely to improve adoption but could potentially be further refined and used to improve sustainability in future work. In addition, the IRPP members agreed to combine the two highly rated strategies related to tailoring recruitment strategies.

**Table 2:** Importance and Feasibility of Implementation Strategies to Increase Extension Agents’ Adoption of Built Environment Approaches

Implementation strategy name and description	Importance	Feasibility
	M (SD)	
Use technology for evaluation* through assistance with gathering impact	4.5 (±.55)	4.5 (±.55)
Create program guide* that details the process of implementing a built environment intervention	4.3 (± 1.21)	4.3 (±.52)
Tailor recruitment strategies* through help creating marketing materials to inform the priority population about built environment approaches	4.3 (±.52)	4.3 (±.52)
Tailor recruitment strategies* through help with creating educational materials to share with local coalitions	4.2 (±.75)	4.5 (±.55)
Conduct demonstration events* through help with implementation and evaluation	4.2 (±.75)	4.3 (±.82)
Build partner relationships* by increasing communication with Montana Department of Public Health and Human Services	3.8 (±.41)	4.5 (±.55)
Increase demand** through leveraging existing physical activity programs to engage community members	3.7 (± 1.37)	4.5 (±.55)
Leverage funding sources* through help with writing a larger grant	3.7 (± 1.51)	4.3 (±.82)
Leverage funding sources* through help obtaining mini-grants	3.7 (± 1.37)	4.3 (±.52)
Conduct local consensus discussions** through facilitation assistance	3.5 (± 1.01)	4.0 (± 1.01)
Provide resources* through increasing communication with Extension Community Vitality Program	2.8 (± 1.60)	4.7 (±.52)
Leverage program champions* through support to identify new program champions and build partnerships	2.3 (± 1.51)	3.8 (± 1.17)

^*^ Strategies from the Implementation Strategies Applied in Communities (ISAC) compilation [14]

^**^ Strategies from the Expert Recommendations for Implementing Change (ERIC) compilation [2]

Following the in-person IRPP meeting, the research team reviewed the remaining three strategies (*create program guide, tailor recruitment strategies, conduct demonstration events*), compared them with the list of barriers, and made final decisions on strategies to include to alleviate each barrier, along with mechanisms of action (hypothesized to lead to increased acceptability, appropriateness, and feasibility of built environment approaches among Agents, which is posited to lead to the primary outcome, increased adoption [67]). Three final changes were made: 1) The highly rated strategies were matched to two of the four identified barriers (community members’ attitudes and access to knowledge and information), but not the remaining two barriers (available resources or communications). Thus, the research team incorporated the highest ranked strategies to alleviate each of these barriers: *leverage funding sources* was selected to address the available resources barrier and *provide resources* was selected to address the communications barrier. 2) The *conduct demonstration events* strategy was expanded to *provide technical assistance* to ensure Agents could receive support on the complete process of changing the built environment, from assessing needs to planning and implementing to evaluating and sharing results. 3) *Tailor recruitment strategies* was included in *create program guide,* as it was determined potentially useful to increase the reach of built environment approaches, but not likely to increase adoption. Thus, three final implementation strategies were selected (Table 3). Following session 1, the research team operationalized and developed descriptions of these strategies in preparation for session 2.

**Table 3:** Implementation strategies selected through ISAC Match, with barriers and mechanisms of action

Implementation Strategy Name	Included Implementation Strategies	Barrier	Mechanisms of Action
**Technical assistance** on the process of implementing built environment approaches, including demonstration events	**Provide technical assistance** for guidance on planning and implementing built environment approaches	Access to knowledge and information	Agents’ knowledge and confidence
	**Conduct demonstration events** through help with implementation and evaluation	Community members'attitudes	Community members'perceptions of acceptability, appropriateness, feasibility of physical activity through walking, biking, rolling to everyday destinations
**Built environment guidebook** detailing the process of implementing a built environment intervention	**Create program guide** that details the process of implementing a built environment intervention	Access to knowledge and information	Agents’ knowledge and confidence
	**Provide resources** to enhance program delivery	Communications	
**Funding support** to secure internal or external funds to test BE approaches through demonstration events or implement BE approaches	**Leverage funding sources** through help with writing a larger grant	Available resources	Agents’ knowledge and confidence
	**Leverage funding sources** through help obtaining mini-grants		

### Step 4. Tailor implementation strategies

In the second IRPP session, five Agents participated in a virtual brainwriting premortem exercise designed to identify pre-implementation barriers and tailor implementation strategies [26, 27]. The research team presented Agents with descriptions of the three final implementation strategies from session 1, as well as the recruitment strategies to be included in the guidebook. Agents were asked to imagine that it was one year in the future and the strategies had failed (e.g., Agents did not participate or use them, or they did participate but the strategies were not effective in increasing adoption of built environment approaches). Agents were asked to consider specific reasons why strategies failed and enter them in their assigned tab of a shared spreadsheet. Next, they were asked to read other Agents’ assigned tabs and add additional ideas that were prompted. Researchers gave Agents time to ask questions, and the process was repeated for the remaining implementation strategies.

The ideas generated by the brainwriting premortem were analyzed through a rapid deductive qualitative approach [23, 26, 68]. First, one of two researchers (SP or ES) reviewed all spreadsheet entries to determine the presence of unique ideas. Duplicated ideas were consolidated to generate a final list of unique ideas. The second researcher (SP or ES) reviewed the first researcher’s coding and noted areas of disagreement, which were resolved through discussion and agreement and consultation with the senior researcher (LB) as needed. Next, to inform tailoring, each unique idea was coded with modules of the FRAME-IS: what is modified, the nature of the modifications, whether modifications are fidelity consistent or inconsistent, the level of the rationale for modification to inform tailoring, and the goal of the modification [50]. Again, coding was completed by SP or ES, checked by the second coder, and resolved with assistance from LB.

Five Agents participated in session 2 and contributed to the brainwriting premortem. A total of 160 ideas were generated: 57 (37%) for *technical assistance*, 39 (24%) for *tailor recruitment strategies*, 34 (21%) for *funding support*, and 29 (18%) for *built environment guidebook.* After condensing duplicate ideas and removing those deemed unclear or not relevant (n = 10, 6%), 48 unique ideas remained: 15 (31%) for *tailor recruitment strategies*, 14 (29%) for *provide technical assistance*, 10 (21%) for *leverage funding sources*, and nine (19%) for *create program guide.* Unique ideas were primarily related to modifying implementation strategy content (n = 36, 75%) and adding elements to strategies (n = 31, 65%). They were fidelity consistent (n = 48, 100%) and mainly informed by implementers (practitioners) (n = 31, 65%). Ideas aimed to improve feasibility (n = 17, 35%) or appropriateness (n = 6, 13%) or proposed a suggestion that was already planned (n = 8, 17%). The research team made final revisions to the implementation strategies based on the premortem data (see Additional file 2 for coding data). These included, for example, adding additional data collection tools, templates, and case studies to the program guide, adding funding timeline information to a spreadsheet of funding opportunities, and clarifying the technical assistance timeline in the protocols being developed.

## Conclusions

The ISAC match process was developed to apply to community settings because of a lack of guidance on rapid, relevant methods for selecting and tailoring implementation strategies to overcome barriers and capitalize on facilitators. Future work is needed to determine whether the ISAC match process is more efficient – and results in more impactful strategies – than time-intensive, comprehensive matching processes such as Implementation Mapping [37]. Strengths of Implementation Mapping include the development of detailed, theory-driven plans to deploy and evaluate implementation strategies [37]. However, the process may not be feasible when time and resources are constrained. In the case study presented here, ISAC Match was completed over a three-month time frame through eight meeting hours, which also included project kickoff activities. Comparing match processes as overarching, multi-component implementation strategies could be accomplished through tracking the time it takes for researchers and practitioners to complete each process and the eventual impacts on targeted implementation outcomes.

Practitioners’ perceptions of the ISAC Match process and the models used to facilitate it (e.g., IRPP) are also needed and should be used to continue to refine ISAC Match to ensure that research and practice contributions are both valued. For example, researchers could use fidelity checklists that include the core components of participatory research (e.g., shared leadership, open communication, mechanisms for resolving conflicts, and trust and cohesion) to self-rate group processes after IRPP meetings [69]. Alternatively, researchers could record IRPP meetings and use rapid deductive analysis to code core components and identify potential areas of improvement, or use brief surveys to understand practitioners’ perceptions of group processes (e.g., trust, dialogue and listening, influence) based on best practices for community-engaged research [70].

One additional consideration for community settings is the preference for a strength-based approach that considers both barriers and facilitators. Existing implementation science models and methods provide limited guidance on how to integrate capitalizing on facilitators into the process. Implementation logic models recommend including both key barriers and key facilitators but lack specific guidance on how to use knowledge of facilitators to select and tailor implementation strategies [71]. Overall, more research is needed on integrating facilitators, and we encourage researchers who use ISAC Match to report this important step so the process can continue to be refined as more evidence is generated. Related, Step 2 (identify existing implementation strategies) is another novel contribution that aligns with a strength-based approach. In the case study reported here, none of the three identified supports were integrated into the final implementation strategies. More work is needed in future studies to differentiate between resources and supports currently being deployed as implementation strategies versus those that are potentially relevant, but not specifically used as implementation strategies.

Finally, while the focus of this article was guidance for research-practice partnerships, we also recognize the need for practitioners to have pragmatic implementation strategy selection processes to strengthen public health practice (e.g., when research partners or grants are not needed or available). As practitioners likely do not have the need, resources, or capacity to use empirical research methods, we recommend a pragmatic process for each of the four steps. We have provided this guidance elsewhere through practitioner-friendly methods: a figure detailing the four steps along with the ISAC compilation and guidance tools are posted at www.centerfornutrition.org. Briefly, we recommend that practitioners first review available information (both peer-reviewed literature, if available, and grey literature) on integration of the EBI of interest. If little is found, practitioners could then use informal processes such as facilitated discussions to understand potential implementation barriers and facilitators or potential implementation outcomes (RE-AIM dimensions) that may prove challenging. Next, practitioners could review existing materials and hold another facilitated discussion to determine which resources and supports are already in place. Practitioners could then use the ISAC guidance tools to select potential implementation strategies, either by barrier level or RE-AIM dimension. Finally, they could tailor potential strategies to fit in their unique setting, considering who will oversee them, the personnel and actions they are designed to influence, and when and how often they will be used.

While the case study presented here describes the use of ISAC Match in one community setting (a state Extension system) and one type of EBI (built environment approaches), the process could be used across diverse community settings and EBIs. Future work is needed across these settings and EBIs to determine whether the strategies selected through ISAC Match are effective in improving adoption, implementation, and maintenance. We encourage researchers and practitioners to report on the use of ISAC Match and build this evidence. The ISAC Match process, and its accompanying ISAC compilation and tools, will need to be updated over time as the use of implementation strategies grows in community settings.

## Supplementary Information

Additional file 1: Implementation strategy importance and feasibility ratings. Raw data of Montana State University Extension Agents’ perceptions of importance and feasibility of 12 potential implementation strategies.

Additional file 2: Brainwriting premortem tailoring data. Description of data: Ideas for tailoring implementation strategies generated through a premortem braining writing session and coded with modules of the FRAME-IS.

## Data Availability

All data generated or analyzed during this study are included in this published article and its supplementary information files.

## Abbreviations

CFIR: Consolidated Framework for Implementation Research
EBIs: Evidence-Based Interventions
ERIC: Expert Recommendation for Implementing Change
FRAME-IS: Framework for Reporting Adaptations and Modifications to Evidence-based Implementation Strategies
ISAC: Implementation Strategies Applied in Communities
IRPP: Integrated Research-Practice Partnership
RE-AIM: Reach, Effectiveness, Adoption, Implementation, Maintenance
